# 2-{4-[Acet­yl(eth­yl)amino]­benzene­sulfonamido}­benzoic acid

**DOI:** 10.1107/S1600536812013864

**Published:** 2012-04-04

**Authors:** Ghulam Mustafa, Islam Ullah Khan, Farhan Mehmood Khan, Mehmet Akkurt

**Affiliations:** a Department of Chemistry, GC University, Lahore 54000, Pakistan; b Department of Physics, Faculty of Sciences, Erciyes University, 38039 Kayseri, Turkey

## Abstract

In the title compound, C_17_H_18_N_2_O_5_S, the dihedral angle between the aromatic rings is 68.59 (10)° and the C—S—N—C torsion angle is −81.84 (18)°. The mol­ecular conformation is stabilized by an intra­molecular N—H⋯O hydrogen bond, generating an *S*(6) ring. In the crystal, mol­ecules are linked by C—H⋯O and O—H⋯O hydrogen bonds into a three-dimensional network.

## Related literature

For related structures and background to the biological properties of sulfonamides, see: Mustafa *et al.* (2010 ▶, 2011 ▶); Khan *et al.* (2011 ▶).
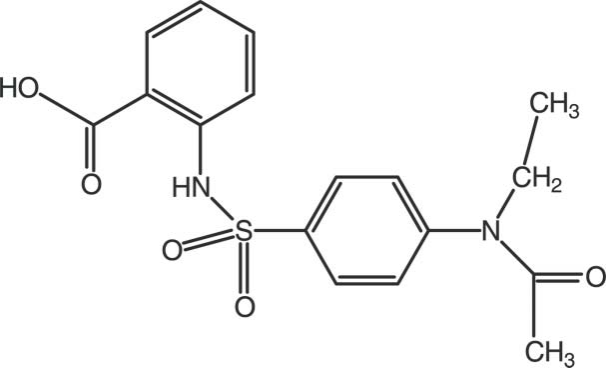


## Experimental

### 

#### Crystal data

C_17_H_18_N_2_O_5_S
*M* *_r_* = 362.40
Orthorhombic, 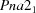

*a* = 18.0371 (7) Å
*b* = 12.0249 (4) Å
*c* = 7.8430 (2) Å
*V* = 1701.10 (10) Å^3^
*Z* = 4
Mo *K*α radiation
μ = 0.22 mm^−1^
*T* = 296 K
0.35 × 0.29 × 0.27 mm

#### Data collection

Bruker APEXII CCD diffractometer
9278 measured reflections
3880 independent reflections
3327 reflections with *I* > 2σ(*I*)
*R*_int_ = 0.040

#### Refinement

*R*[*F* ^2^ > 2σ(*F* ^2^)] = 0.035
*wR*(*F* ^2^) = 0.093
*S* = 0.99
3880 reflections
233 parameters
2 restraints
H atoms treated by a mixture of independent and constrained refinement
Δρ_max_ = 0.35 e Å^−3^
Δρ_min_ = −0.18 e Å^−3^
Absolute structure: Flack (1983 ▶), 1612 Freidel pairs
Flack parameter: 0.05 (6)

### 

Data collection: *APEX2* (Bruker, 2007 ▶); cell refinement: *SAINT* (Bruker, 2007 ▶); data reduction: *SAINT*; program(s) used to solve structure: *SHELXS97* (Sheldrick, 2008 ▶); program(s) used to refine structure: *SHELXL97* (Sheldrick, 2008 ▶); molecular graphics: *ORTEP-3 for Windows* (Farrugia, 1997 ▶) and *PLATON* (Spek, 2009 ▶); software used to prepare material for publication: *WinGX* (Farrugia, 1999 ▶) and *PLATON*.

## Supplementary Material

Crystal structure: contains datablock(s) global, I. DOI: 10.1107/S1600536812013864/hb6720sup1.cif

Structure factors: contains datablock(s) I. DOI: 10.1107/S1600536812013864/hb6720Isup2.hkl

Supplementary material file. DOI: 10.1107/S1600536812013864/hb6720Isup3.cml

Additional supplementary materials:  crystallographic information; 3D view; checkCIF report

## Figures and Tables

**Table 1:** Hydrogen-bond geometry (Å, °)

*D*—H⋯*A*	*D*—H	H⋯*A*	*D*⋯*A*	*D*—H⋯*A*
O1—H1⋯O5^i^	0.82	1.82	2.599 (2)	158
N1—H1*N*⋯O2	0.85 (2)	1.93 (2)	2.627 (2)	138 (2)
C12—H12⋯O3^ii^	0.93	2.45	3.356 (2)	164
C15—H15*C*⋯O3^ii^	0.96	2.53	3.400 (3)	150
C17—H17*A*⋯O2^ii^	0.96	2.58	3.486 (4)	158

Symmetry codes: (i) 
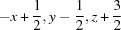
; (ii) 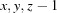.

**Fig. 1.**
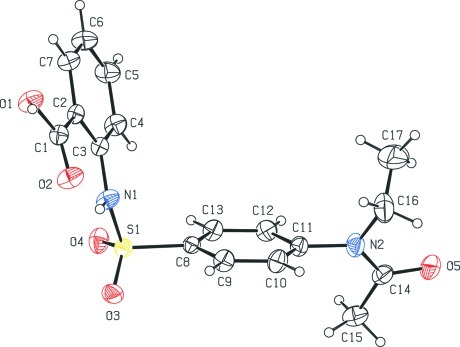
The molecular structure of the title compound, showing displacement ellipsoids drawn at the 30% probability level.

**Fig. 2.**
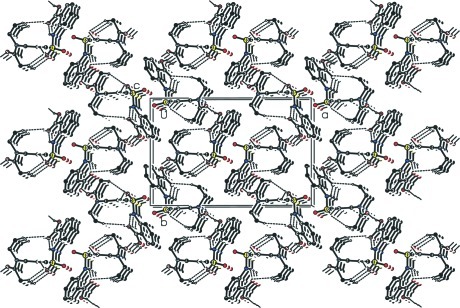
View of the packing diagram and the hydrogen bonding of (I) along the c axis. Hydrogen atoms that not involved in the hydrogen-bonding (dashed lines) have been omitted for clarity.

## supplementary crystallographic information

### Comment

As part of our ongoing studies of sulfonamides with potential biological properties (Mustafa *et al.*, 2010, 2011; Khan *et al.*, 2011), we now describe the title compound, (I).

In the title compound (I), (Fig. 1), the C2—C7 benzene ring makes a dihedral angle of 68.59 (10)° with the C8—C13 benzene ring. All the bond lengths are similar to those of the related molecules (Mustafa *et al.*, 2010; 2011; Khan *et al.*, 2011). The torsion angles C3—N1—S1—C8, C11—N2—C16—C17 and C11—N2—C14—C15 are -81.84 (18), 94.2 (3), and 7.0 (3)°, respectively.

The molecular conformation of (I) is stabilized by intramolecular N—H···O hydrogen-bond interaction (Table 1), which generate one S(6) ring. In the crystal, molecules are linked by C—H···O and O—H···O hydrogen bonds to form a three- dimensional network structure (Table 1, Fig. 2).

### Experimental

To an aquious solution of *p*-amino benzoic acid (1.0 g, 7.3 mmol), sodium carbonate (1 N) was added to adjust the pH 8. Then added 4-(acetylamino) benzenesulfonyl chloride (2.21 g, 9.48 mmol) and the mixture was stirred at room temperature keeping the pH of the mixture up to 8.0 with occasional addition of sodium carbonate solution. Progress and completion of the reaction was confirmed by TLC and conversion of suspension into clear solution. After 2 h, whole mixture was poured into a beaker and the pH was adjusted to 2.0 by 1 N HCl. Pprecipitates were produced which were filtered and washed with distilled water. The prepared sulfonamide (2-({[4-(acetylamino)phenyl]sulfonyl}amino) benzoic acid) (1.0 g, 3 mmol), DMF (10 ml) and n-hexane washed sodium hydride (0.22 g, 9.0 mmol) were stirred at room temperature for 40 min followed by the addition of ethyl iodide (0.61 g, 3.9 mmol). The whole reaction mixture was stirred till the completion of the reaction and poured into crushed ice in a beaker. The pH of the mixture was adjusted to 4.0 with 1 N HCl. Precipitates were produced, filtered and washed twice with distilled water and crystallized from chloroform solution as brown blocks.

### Refinement

The NH H atom was located in a difference map and refined with the distance restraint N—H = 0.86 (2) Å freely. The remaining H atoms were placed in calculated positions [O–H = 0.82 Å, C—H = 0.93, 0.96 and 0.97 Å], with *U*_iso_(H) = 1.5*U*_eq_(C, O) for the methyl and hydroxyl H atoms and *U*_iso_(H) = 1.2*U*_eq_(C) for the other H atoms. Four reflections giving bad agreements with Fc, *viz*. (200), (110), (011) and (122), were omitted during the final cycles of refinement.

### Crystal data

**Table:**

C_17_H_18_N_2_O_5_S	*F*(000) = 760
*M**_r_* = 362.40	*D*_x_ = 1.415 Mg m^−^^3^
Orthorhombic, *P**n**a*2_1_	Mo *K*α radiation, λ = 0.71073 Å
Hall symbol: P 2c -2n	Cell parameters from 4557 reflections
*a* = 18.0371 (7) Å	θ = 2.3–28.2°
*b* = 12.0249 (4) Å	µ = 0.22 mm^−^^1^
*c* = 7.8430 (2) Å	*T* = 296 K
*V* = 1701.10 (10) Å^3^	Block, brown
*Z* = 4	0.35 × 0.29 × 0.27 mm

### Data collection

**Table:**

Bruker APEXII CCD diffractometer	3327 reflections with *I* > 2σ(*I*)
Radiation source: sealed tube	*R*_int_ = 0.040
Graphite monochromator	θ_max_ = 28.3°, θ_min_ = 2.8°
φ and ω scans	*h* = −24→19
9278 measured reflections	*k* = −13→16
3880 independent reflections	*l* = −10→10

### Refinement

**Table:**

Refinement on *F*^2^	Secondary atom site location: difference Fourier map
Least-squares matrix: full	Hydrogen site location: inferred from neighbouring sites
*R*[*F*^2^ > 2σ(*F*^2^)] = 0.035	H atoms treated by a mixture of independent and constrained refinement
*wR*(*F*^2^) = 0.093	*w* = 1/[σ^2^(*F*_o_^2^) + (0.0604*P*)^2^] where *P* = (*F*_o_^2^ + 2*F*_c_^2^)/3
*S* = 0.99	(Δ/σ)_max_ < 0.001
3880 reflections	Δρ_max_ = 0.35 e Å^−^^3^
233 parameters	Δρ_min_ = −0.18 e Å^−^^3^
2 restraints	Absolute structure: Flack (1983), 1612 Freidel pairs
Primary atom site location: structure-invariant direct methods	Flack parameter: 0.05 (6)

### Special details

**Table:**

Geometry. Bond distances, angles *etc*. have been calculated using the rounded fractional coordinates. All su's are estimated from the variances of the (full) variance-covariance matrix. The cell e.s.d.'s are taken into account in the estimation of distances, angles and torsion angles
Refinement. Refinement on *F*^2^ for ALL reflections except those flagged by the user for potential systematic errors. Weighted *R*-factors *wR* and all goodnesses of fit *S* are based on *F*^2^, conventional *R*-factors *R* are based on *F*, with *F* set to zero for negative *F*^2^. The observed criterion of *F*^2^ > σ(*F*^2^) is used only for calculating -*R*-factor-obs *etc*. and is not relevant to the choice of reflections for refinement. *R*-factors based on *F*^2^ are statistically about twice as large as those based on *F*, and *R*-factors based on ALL data will be even larger.

### Fractional atomic coordinates and isotropic or equivalent isotropic displacement parameters (Å^2^)

**Table:**

	*x*	*y*	*z*	*U*_iso_*/*U*_eq_	
S1	0.09768 (2)	1.03872 (4)	1.00764 (6)	0.0403 (1)	
O1	0.06408 (9)	0.65010 (13)	1.42241 (19)	0.0555 (5)	
O2	0.12860 (8)	0.80244 (12)	1.3690 (2)	0.0570 (5)	
O3	0.13309 (9)	1.10513 (13)	1.13429 (18)	0.0551 (5)	
O4	0.02644 (8)	1.06853 (14)	0.9452 (2)	0.0568 (5)	
O5	0.36221 (10)	1.08713 (16)	0.1903 (2)	0.0660 (6)	
N1	0.09488 (10)	0.91519 (15)	1.0928 (2)	0.0473 (6)	
N2	0.30964 (11)	1.02317 (15)	0.4272 (3)	0.0544 (6)	
C1	0.08374 (10)	0.73379 (15)	1.3240 (2)	0.0402 (5)	
C2	0.04690 (9)	0.73281 (15)	1.1545 (2)	0.0376 (5)	
C3	0.05278 (10)	0.82314 (14)	1.0418 (2)	0.0371 (5)	
C4	0.01818 (12)	0.81716 (18)	0.8837 (3)	0.0494 (6)	
C5	−0.02159 (13)	0.72412 (18)	0.8387 (3)	0.0569 (8)	
C6	−0.02717 (13)	0.63543 (19)	0.9473 (3)	0.0584 (7)	
C7	0.00674 (11)	0.63937 (17)	1.1041 (3)	0.0486 (6)	
C8	0.15845 (10)	1.03090 (13)	0.8330 (2)	0.0347 (5)	
C9	0.23390 (11)	1.02380 (17)	0.8644 (3)	0.0461 (6)	
C10	0.28288 (12)	1.02284 (18)	0.7301 (3)	0.0497 (7)	
C11	0.25724 (11)	1.02912 (16)	0.5651 (2)	0.0428 (6)	
C12	0.18157 (11)	1.03649 (16)	0.5338 (2)	0.0456 (6)	
C13	0.13254 (11)	1.03771 (17)	0.6682 (2)	0.0428 (6)	
C14	0.32075 (12)	1.10446 (17)	0.3124 (3)	0.0479 (6)	
C15	0.28352 (13)	1.21419 (17)	0.3350 (4)	0.0609 (8)	
C16	0.35281 (15)	0.9186 (2)	0.4124 (4)	0.0765 (10)	
C17	0.3174 (2)	0.8394 (3)	0.2929 (7)	0.1147 (17)	
H1	0.09620	0.63950	1.49470	0.0830*	
H1N	0.1178 (11)	0.9097 (19)	1.187 (2)	0.047 (6)*	
H4	0.02200	0.87640	0.80800	0.0590*	
H5	−0.04500	0.72140	0.73310	0.0680*	
H6	−0.05380	0.57270	0.91500	0.0700*	
H7	0.00280	0.57890	1.17760	0.0580*	
H9	0.25130	1.01970	0.97580	0.0550*	
H10	0.33350	1.01790	0.75080	0.0600*	
H12	0.16410	1.04060	0.42240	0.0550*	
H13	0.08190	1.04310	0.64770	0.0510*	
H15A	0.31060	1.27030	0.27400	0.0910*	
H15B	0.28220	1.23280	0.45390	0.0910*	
H15C	0.23380	1.21010	0.29150	0.0910*	
H16A	0.40250	0.93560	0.37260	0.0920*	
H16B	0.35700	0.88430	0.52390	0.0920*	
H17A	0.27050	0.81600	0.33830	0.1720*	
H17B	0.34890	0.77570	0.27820	0.1720*	
H17C	0.30990	0.87500	0.18470	0.1720*	

### Atomic displacement parameters (Å^2^)

**Table:**

	*U*^11^	*U*^22^	*U*^33^	*U*^12^	*U*^13^	*U*^23^
S1	0.0487 (2)	0.0385 (2)	0.0338 (2)	−0.0018 (2)	0.0058 (2)	0.0024 (2)
O1	0.0657 (9)	0.0549 (8)	0.0460 (8)	−0.0130 (7)	−0.0115 (7)	0.0178 (7)
O2	0.0614 (9)	0.0576 (8)	0.0520 (8)	−0.0156 (7)	−0.0192 (7)	0.0128 (7)
O3	0.0783 (10)	0.0481 (8)	0.0388 (7)	−0.0115 (7)	0.0075 (7)	−0.0068 (6)
O4	0.0479 (8)	0.0636 (9)	0.0590 (9)	0.0107 (7)	0.0093 (7)	0.0114 (8)
O5	0.0780 (12)	0.0699 (11)	0.0501 (9)	−0.0151 (8)	0.0208 (8)	0.0087 (8)
N1	0.0584 (11)	0.0472 (9)	0.0363 (9)	−0.0136 (8)	−0.0088 (8)	0.0139 (8)
N2	0.0615 (11)	0.0523 (10)	0.0495 (10)	0.0049 (8)	0.0198 (9)	0.0117 (9)
C1	0.0401 (9)	0.0410 (9)	0.0394 (9)	0.0035 (7)	−0.0001 (7)	0.0065 (8)
C2	0.0317 (8)	0.0421 (9)	0.0390 (9)	0.0000 (7)	0.0032 (7)	0.0026 (8)
C3	0.0350 (9)	0.0390 (8)	0.0374 (10)	−0.0010 (7)	0.0016 (7)	0.0016 (7)
C4	0.0558 (12)	0.0521 (11)	0.0402 (10)	−0.0040 (9)	−0.0063 (9)	0.0083 (9)
C5	0.0664 (14)	0.0582 (13)	0.0462 (12)	−0.0054 (10)	−0.0154 (10)	−0.0039 (10)
C6	0.0641 (13)	0.0492 (12)	0.0618 (13)	−0.0110 (10)	−0.0135 (11)	−0.0042 (10)
C7	0.0503 (11)	0.0422 (10)	0.0534 (12)	−0.0061 (8)	−0.0039 (9)	0.0050 (9)
C8	0.0404 (10)	0.0322 (8)	0.0315 (8)	−0.0021 (6)	0.0018 (7)	0.0012 (7)
C9	0.0462 (11)	0.0583 (12)	0.0339 (9)	−0.0017 (9)	−0.0074 (8)	0.0039 (9)
C10	0.0383 (11)	0.0615 (12)	0.0494 (12)	−0.0003 (9)	−0.0015 (9)	0.0063 (10)
C11	0.0455 (11)	0.0425 (10)	0.0405 (10)	−0.0007 (7)	0.0081 (8)	0.0031 (8)
C12	0.0526 (11)	0.0530 (11)	0.0312 (10)	−0.0037 (9)	−0.0065 (8)	0.0027 (8)
C13	0.0378 (10)	0.0531 (11)	0.0376 (10)	−0.0022 (8)	−0.0065 (8)	0.0040 (8)
C14	0.0513 (11)	0.0468 (10)	0.0457 (11)	−0.0119 (8)	0.0002 (9)	0.0054 (9)
C15	0.0686 (15)	0.0468 (11)	0.0673 (15)	−0.0098 (10)	−0.0047 (12)	0.0065 (11)
C16	0.0816 (19)	0.0763 (17)	0.0716 (17)	0.0207 (14)	0.0323 (14)	0.0162 (15)
C17	0.117 (3)	0.0541 (16)	0.173 (4)	−0.0027 (17)	0.033 (3)	−0.008 (2)

### Geometric parameters (Å, º)

**Table:**

S1—O3	1.4256 (16)	C9—C10	1.375 (3)
S1—O4	1.4211 (15)	C10—C11	1.376 (3)
S1—N1	1.6295 (18)	C11—C12	1.390 (3)
S1—C8	1.7568 (17)	C12—C13	1.376 (2)
O1—C1	1.317 (2)	C14—C15	1.491 (3)
O2—C1	1.209 (2)	C16—C17	1.481 (5)
O5—C14	1.233 (3)	C4—H4	0.9300
O1—H1	0.8200	C5—H5	0.9300
N1—C3	1.401 (2)	C6—H6	0.9300
N2—C14	1.344 (3)	C7—H7	0.9300
N2—C16	1.484 (3)	C9—H9	0.9300
N2—C11	1.438 (3)	C10—H10	0.9300
N1—H1N	0.849 (17)	C12—H12	0.9300
C1—C2	1.486 (2)	C13—H13	0.9300
C2—C3	1.404 (2)	C15—H15A	0.9600
C2—C7	1.394 (3)	C15—H15B	0.9600
C3—C4	1.390 (3)	C15—H15C	0.9600
C4—C5	1.375 (3)	C16—H16A	0.9700
C5—C6	1.369 (3)	C16—H16B	0.9700
C6—C7	1.374 (3)	C17—H17A	0.9600
C8—C9	1.386 (3)	C17—H17B	0.9600
C8—C13	1.377 (2)	C17—H17C	0.9600
			
O3—S1—O4	120.26 (10)	O5—C14—C15	121.0 (2)
O3—S1—N1	103.82 (9)	N2—C14—C15	119.8 (2)
O3—S1—C8	107.08 (9)	N2—C16—C17	111.6 (2)
O4—S1—N1	110.07 (9)	C3—C4—H4	120.00
O4—S1—C8	108.00 (9)	C5—C4—H4	120.00
N1—S1—C8	106.87 (8)	C4—C5—H5	120.00
C1—O1—H1	109.00	C6—C5—H5	120.00
S1—N1—C3	128.33 (13)	C5—C6—H6	120.00
C11—N2—C16	116.48 (19)	C7—C6—H6	120.00
C14—N2—C16	119.1 (2)	C2—C7—H7	120.00
C11—N2—C14	124.45 (18)	C6—C7—H7	120.00
C3—N1—H1N	116.8 (15)	C8—C9—H9	120.00
S1—N1—H1N	114.4 (15)	C10—C9—H9	120.00
O1—C1—C2	113.44 (16)	C9—C10—H10	120.00
O1—C1—O2	122.08 (16)	C11—C10—H10	120.00
O2—C1—C2	124.47 (16)	C11—C12—H12	120.00
C1—C2—C3	121.54 (15)	C13—C12—H12	120.00
C1—C2—C7	119.49 (17)	C8—C13—H13	120.00
C3—C2—C7	118.96 (16)	C12—C13—H13	120.00
C2—C3—C4	119.18 (16)	C14—C15—H15A	109.00
N1—C3—C2	118.19 (15)	C14—C15—H15B	109.00
N1—C3—C4	122.62 (16)	C14—C15—H15C	109.00
C3—C4—C5	120.4 (2)	H15A—C15—H15B	109.00
C4—C5—C6	120.8 (2)	H15A—C15—H15C	109.00
C5—C6—C7	119.8 (2)	H15B—C15—H15C	110.00
C2—C7—C6	120.85 (19)	N2—C16—H16A	109.00
C9—C8—C13	120.26 (17)	N2—C16—H16B	109.00
S1—C8—C13	121.13 (14)	C17—C16—H16A	109.00
S1—C8—C9	118.52 (14)	C17—C16—H16B	109.00
C8—C9—C10	119.7 (2)	H16A—C16—H16B	108.00
C9—C10—C11	120.3 (2)	C16—C17—H17A	109.00
N2—C11—C10	118.92 (19)	C16—C17—H17B	109.00
N2—C11—C12	121.05 (16)	C16—C17—H17C	109.00
C10—C11—C12	119.98 (17)	H17A—C17—H17B	109.00
C11—C12—C13	119.78 (15)	H17A—C17—H17C	110.00
C8—C13—C12	120.02 (18)	H17B—C17—H17C	109.00
O5—C14—N2	119.2 (2)		
			
O3—S1—N1—C3	165.17 (17)	O2—C1—C2—C3	−10.4 (3)
O4—S1—N1—C3	35.20 (19)	O1—C1—C2—C7	−10.4 (2)
C8—S1—N1—C3	−81.84 (18)	C1—C2—C7—C6	−179.19 (19)
N1—S1—C8—C13	109.88 (16)	C7—C2—C3—C4	0.3 (3)
N1—S1—C8—C9	−73.62 (16)	C7—C2—C3—N1	−178.43 (17)
O3—S1—C8—C9	37.13 (17)	C1—C2—C3—C4	178.99 (17)
O4—S1—C8—C9	167.99 (15)	C3—C2—C7—C6	−0.5 (3)
O3—S1—C8—C13	−139.37 (15)	C1—C2—C3—N1	0.3 (2)
O4—S1—C8—C13	−8.52 (18)	C2—C3—C4—C5	0.3 (3)
S1—N1—C3—C2	−166.53 (14)	N1—C3—C4—C5	178.98 (19)
S1—N1—C3—C4	14.8 (3)	C3—C4—C5—C6	−0.8 (3)
C14—N2—C11—C12	64.0 (3)	C4—C5—C6—C7	0.6 (4)
C16—N2—C14—C15	−174.0 (2)	C5—C6—C7—C2	0.0 (3)
C16—N2—C11—C10	62.2 (3)	S1—C8—C9—C10	−176.96 (16)
C11—N2—C14—C15	7.0 (3)	C9—C8—C13—C12	0.6 (3)
C14—N2—C16—C17	−85.0 (3)	C13—C8—C9—C10	−0.4 (3)
C11—N2—C16—C17	94.2 (3)	S1—C8—C13—C12	177.02 (15)
C11—N2—C14—O5	−173.6 (2)	C8—C9—C10—C11	0.1 (3)
C14—N2—C11—C10	−118.7 (2)	C9—C10—C11—C12	0.0 (3)
C16—N2—C14—O5	5.5 (3)	C9—C10—C11—N2	−177.39 (19)
C16—N2—C11—C12	−115.1 (2)	C10—C11—C12—C13	0.1 (3)
O2—C1—C2—C7	168.24 (18)	N2—C11—C12—C13	177.48 (18)
O1—C1—C2—C3	170.93 (16)	C11—C12—C13—C8	−0.4 (3)

### Hydrogen-bond geometry (Å, º)

**Table:**

*D*—H···*A*	*D*—H	H···*A*	*D*···*A*	*D*—H···*A*
O1—H1···O5^i^	0.82	1.82	2.599 (2)	158
N1—H1*N*···O2	0.85 (2)	1.93 (2)	2.627 (2)	138 (2)
C12—H12···O3^ii^	0.93	2.45	3.356 (2)	164
C15—H15*C*···O3^ii^	0.96	2.53	3.400 (3)	150
C17—H17*A*···O2^ii^	0.96	2.58	3.486 (4)	158

Symmetry codes: (i) −*x*+1/2, *y*−1/2, *z*+3/2; (ii) *x*, *y*, *z*−1.
